# Clinical Yield of Colonoscopy in Evaluation of Young Women with Constipation: An Age- and Gender-Based Analysis

**DOI:** 10.3390/diagnostics15101209

**Published:** 2025-05-11

**Authors:** Amani Beshara, Avraham Yitzhak, Revital Guterman, Ruhama Elhayany, Majd Khader, Sarah Weissmann, Naim Abu-Freha

**Affiliations:** 1 Hillel Yaffe Medical Center, Hadera 3820302, Israel; 2 Rappaport Faculty of Medicine, Technion—Israel Institute of Technology, Haifa 3525433, Israel; 3 Assuta Medical Center, Beer-Sheva 8489507, Israel; 4 Faculty of Health Sciences, Ben Gurion University of the Negev, Beer-Sheva 8410501, Israel

**Keywords:** constipation, young women, colonoscopy, inflammatory bowel disease, diverticulosis, colorectal cancer

## Abstract

**Background:** Constipation is one of the most common gastrointestinal complaints among women, with a variety of contributing factors. We aimed to assess the role of colonoscopy in evaluating young women with constipation. **Methods:** A multi-center, large cohort, retrospective study included all data from colonoscopies performed between 2016 and 2023 in seven endoscopy departments. The indications and findings of the procedures were collected, and findings of young women aged ≤40 y with constipation as an indication were compared to older women and men of the same age groups. **Results:** The cohort comprised 377,795 patients, including 198,629 (52.6%) females and 179,166 (47.4%) males. In total, 7872 females underwent colonoscopy for constipation and other indications **(Cohort 1)**. In addition, 6852 women were referred for a colonoscopy for constipation only **(Cohort 2)**. In sum, 75% of colonoscopies in women <40 y were normal in both cohorts. In **Cohort 1,** inflammatory bowel diseases (IBD) were significantly higher in women <40 y with Ulcerative Colitis (UC) (1.2%) and Crohn’s disease (CD) (0.7%), *p* < 0.001). The rate of IBD was lower but still significant in **Cohort 2**. In both cohorts, diverticulosis and polyp rates exponentially increased with age >40 y, *p* < 0.001. Higher rates of diverticulosis and polyps were found among males <40 y in **Cohort 1.** One case (0.1%) of Colorectal cancer (CRC) was found in <40 y women. Similar IBD and CRC rates were found in males and females of all ages, *p* > 0.05. **Conclusions:** The diagnostic yield of colonoscopy for investigating isolated constipation in young females is not significant. Diagnostic work-up should be guided by accurate clinical understanding.

## 1. Introduction

Constipation is one of the most common complaints of the gastrointestinal tract [1], characterized by unsatisfactory defecation as a result of infrequent stools, difficult stool passage, or both [2,3]. The prevalence of constipation is estimated to be about 16% and varies between 0.7 and 79.0% in different regions worldwide [2]. Chronic constipation tends to be more common in women than men and increases with age [1,2,3]. By some estimates, it is more common in women by a 2:1 margin [2]. It is still not fully understood why women are more susceptible, but it may be due to a variety of factors: sexual hormones which fluctuate during menstruation, pregnancy, and menopause can influence gastrointestinal motility and function. These hormonal changes may impact the frequency and severity of symptoms like constipation, bloating, or abdominal discomfort, making women more prone to these conditions at various life stages. Addition factors include sociologically derived mental stress and problems with straining related to pelvic floor muscle dysfunction [3]. The higher prevalence of symptoms in women could also result from the fact that women have a higher tendency to report their physical symptoms [4]. The pathophysiology of constipation is multifactorial [5]. Constipation is commonly grouped into primary and secondary causes [6,7]. Primary causes are functional intrinsic problems of colonic or anorectal function (low transit time or outlet dysfunction), whereas secondary causes are related to organic disease, systemic disease, or medications including inadequate fluid intake, metabolic disturbances, neurological disorders, myopathic disorders, and structural abnormalities [8,9,10,11,12]. Functional constipation is a common functional bowel disorder. Its prevalence is estimated between 2 and 28% of the population and varies between countries. In a survey that included 2012 Israeli participants, 36.4% met the diagnostic criteria for at least one gastrointestinal functional disorder, while 30.8% did so for any bowel disorder. The rates were higher for women associated negatively with psychosocial variables and health utilization [13]. Significant differences in indications and findings of colonoscopies in females compared to males with bowel functional disorder were reported in another multicenter Israeli study in which the indication of change in bowel habits was significantly higher in females, while more colonic pathologies were observed in males [14]. Environmental, educational, cultural, ethnic, dietary, and genetic factors can influence the reporting of symptoms. Individuals from different cultural or ethnic backgrounds may have varying perceptions of health and illness which can affect their willingness to report symptoms. Educational background can also impact one’s understanding of symptoms, leading to either underreporting or over-reporting depending on the level of awareness. Additionally, dietary habits can directly influence gastrointestinal health, while genetic predispositions may make certain populations more prone to specific conditions, further complicating symptom reporting. These factors can interact in complex ways, contributing to variability in symptom presentation and reporting across diverse populations [2,15,16,17,18]. Functional constipation presents without organic lesions or physiological abnormalities and is diagnosed as per the Rome IV criteria [17]. The Rome IV criteria have been refined to consider constipation as a feature of several distinct but occasionally overlapping disorders of gut–brain interaction. This continuum includes functional constipation, irritable bowel syndrome (IBS) with constipation, opioid-induced constipation, and functional defecation disorders, all of which result in problematic and unsatisfactory defecation [15,17,18]. Most of these functional disorders are more common in women [4,18]. In some cohorts, functional constipation was reported in up to 28.8% of healthy women (average age 37 years) [7,10]. Investigating constipation should be clinically directed depending on accurate clinical history, physical examination, and laboratory indices. Structural evaluation of the colon should be recommended if the patient has alarm signs (anemia, weight loss, abdominal pain, and hematochezia), has a new onset of constipation or is older than the screening age for colonoscopy (>50) and has not undergone previous screening for colorectal cancer [19]. The American Society for Gastrointestinal Endoscopy (ASGE) published guidelines in 2014 on the use of endoscopy in the management of constipation. Accordingly, the clinical utility of colonoscopy in patients with isolated constipation is low and is comparable with asymptomatic patients who undergo colonoscopy for colorectal cancer screening [19]. Although functional constipation is very common among young women as mentioned previously, colonoscopy continues to be a commonly used diagnostic tool for patients presenting with constipation, including many young women who may have functional constipation. While it is an important diagnostic tool for certain gastrointestinal disorders, there are many studies evaluating the suitability of colonoscopy in patients presenting with constipation, but the role of colonoscopy in young females with constipation as a sole complaint has not been studied. Many physicians, perhaps due to the desire for comprehensive evaluation, continue to refer young women with isolated constipation for colonoscopy. However, this practice may not be necessary, especially when the symptoms are consistent with functional constipation. In this study, we aim to evaluate the role of colonoscopy in diagnosing isolated constipation in young women, exploring whether it is an effective diagnostic tool or whether alternative approaches could be more appropriate for managing this common gastrointestinal condition. Understanding the necessity and utility of colonoscopy in this context could lead to more efficient, cost-effective, and patient-centered approaches in the management of functional constipation.

## 2. Materials and Methods

### 2.1. Study Population

We conducted a multi-center, large cohort, cross-sectional retrospective study of colonoscopies performed between the years 2016 and 2023. Only the first colonoscopy was included in the study. Otherwise, all performed colonoscopies in an outpatient setting were included in the study.

### 2.2. Data Collection

Data were collected from seven Assuta Medical Center endoscopy departments located throughout Israel. Data were extracted from medical records and electronic charts using the platform “MdClone”. Demographic information, indications for colonoscopy, preparation quality, cecal intubation, and the colonoscopy findings were retrieved for each patient. We included only patients with constipation as indications for colonoscopy, subdivided as those underwent colonoscopy for constipation and other indications (Cohort 1), and those referred for colonoscopy for constipation only (Cohort 2). There were 1305 (10%) procedures excluded because of poor preparation and incomplete examination. Poor preparation was defined as a Boston bowel preparation scale score of less than 5. The indications and findings were compared between males and females. Data regarding comorbidities and co-indications among females referred to colonoscopy because of constipation were collected, and in addition the findings of colonoscopies were compared between the different age groups and between females and males.

### 2.3. Statistical Analysis

Patient characteristics were presented as mean ± SD for continuous variables and as percentages for categorical variables. Categorical variables were compared using the chi-square test. Continuous variables were examined with the student *t*-test. All statistical analyses were performed using IBM SPSS version 29 (Chicago, IL, USA). *p*-values less than 0.05 were considered statistically significant. The study protocol was approved by the Institutional Helsinki Committee of Assuta Medical Centers (approval number 10-22 date 28 March 2022). Informed consent was waived due to the retrospective non-interventional study design.

## 3. Results

A cohort of 377,795 patients underwent colonoscopies between the years 2016 and 2023. In total, 198,629 females (52.6%) and 179,166 males (47.4%) were included. Of these, 7872 females underwent colonoscopy for constipation and other indications as presented in Table 1. Thirty-two percent of them were under the age of 50 years. In addition, 6852 women were referred for colonoscopy for constipation alone. Younger patients had lower rates of metabolic and cardiopulmonary comorbidities but had more associated symptoms, abdominal pain, and rectal bleeding, in comparison to patients older than 50 years of age (Table 1). Patients of older ages, however, had significantly higher rates of positive fecal occult blood test (FOBT) (Table 1).

### 3.1. Cohort 1: Colonoscopy Findings for Constipation and Other Indications

Of the 7872 females who were referred for colonoscopy for constipation among other symptoms, 1017 (13%) were referred at the ages of 18–39 years, 1535 (19.5%) between the ages of 40 and 49 years, 1972 (25%) between 50 and 59 years, and 3348 (42.5%) patients were referred over 60 years. Normal colonoscopy was reported in 761 (74.8%) patients aged 18–39 years, 997 (65%) of the patients aged 40–49 years, 1102 (56%) of the patients aged 50–59 years, and in 1296 (38.7%) patients older than 60 years, *p* < 0.001 (Table 2). UC and CD were more prevalent in young patients under 40 years than in older patients (12 (1.2%) and 7 (0.7%) respectively, *p* < 0.001). Diverticulosis and polyp rates exponentially increased with advanced age (Table 2). The increase was most remarkable between patients aged 40 to 49 years compared to patients under 40 years. Diverticulosis was found in 5 (0.5%) patients in the youngest group and 47 (3.1%) patients in the group 40–49 years, *p* < 0.001. The rate continued to increase in patients of older ages, *p* < 0.001. Colonic polyps were found in 75 (7.4%) patients in the group aged under 40 years vs. 247 (16%) patients aged 40–49 years, *p* < 0.001, and they continued to increase with age, *p* < 0.001. The majority of cases with polyps across all age groups involved a single polyp (61.6%) (Table 2). Similarly, among young females under 40, most detected polyps were also single polyps (70.6%). No significant differences in CRC rates were found between the different groups, *p* = 0.103 (Table 2).

#### Colonoscopy Findings in Males vs. Females

In total, 7872 women and 4038 men older than 18 years underwent colonoscopy for constipation among other indications. Normal colonoscopies were reported among 4156 (52.8%) females versus 1687 (41.8%) males, *p* < 0.001 (Table S1). A similar prevalence of IBD was found in both groups among patients of all ages. UC was seen in 24 (0.3%) females and 12 (0.3%) males, *p* = 0.942. CD was found in 10 (0.1%) females compared to 4 (0.1%) males, *p* = 0.67. No difference was found in IBD or CD prevalence among males and females aged 18–39 years (*p* = 0.6 for UC and *p* = 0.5 for CD) (Table 3). A higher rate of diverticulosis was found in males compared to females: 656 (16.2%) and 915 (11.6%) respectively, *p* < 0.01 (Table S1). This higher prevalence was still significant in the cohort younger than 40 years (seven (1.5%) of the young men vs. five (0.5%) of the young women, *p* < 0.048) (Table 3). Colonic polyps were also found to be higher among males of all ages: 1417 (35.1%) males in comparison to 1189 females (24%), *p* < 0.001) (Table S1). Interestingly, significantly higher rates of polyps were found in young males under 40 years than young females of the same age group (54 (11.3%) vs. 75 (7.4%), *p* = 0.01, respectively) (Table 3). Similar prevalence of CRC was found in both groups of all ages (30 (0.4%) females, 18 (0.4%) males, *p* = 0.6) (Table S1). No significant difference was found in CRC prevalence among patients aged under 40 years, *p* = 0.5 (Table 3).

### 3.2. Cohort 2: Colonoscopy Findings for Isolated Constipation

Of 6852 females, 807 (11.8%) were aged 18–39 years, 1328 (19.4%) were 40–49 years old, 1739 (25.4%) were 50–59 years old, and 2978 (43.5%) were older than 60 years (Table 4). Normal colonoscopy was reported in 608 (75.3%) patients aged 18–39 years, in 863 (65%) patients aged 40–49 years, in 972 (56%) patients aged 50–59 years, and in 1170 (39.3%) patients over 60 years of age, *p* < 0.001 (Table 4). Lower rates of IBD were found in this cohort compared to cohort 1. UC was found to be most prevalent in the 18–39 year-old cohort (six (0.7%) patients), compared to four (0.3%) in the 40–49 year cohort, two (0.1%) in the 50–59 year cohort, and four (0.1%) in the greater than 60-year cohort, *p* = 0.001. The prevalence was not significantly different between the under 40 and 40–49 year groups, *p* = 0.147. CD was found to be less common than UC in all age groups with isolated constipation (18–39 years = 2 (0.2%), 40–49 years = 3 (0.2%), 50–59 years = 0, ≥60 years = 0, *p* = 0.011) (Table 4). As in the cohort with constipation and other indications, the prevalence of diverticulosis and polyps significantly steadily increased with age *p* < 0.001. No statistically significant difference was found between the two cohorts in diverticulosis and polyp rates. A higher rate of diverticulosis was found in the age group 40–49 years compared to the 18–39-year group (35 (2.6%) patients and 3 (0.4%) patients, respectively, *p* < 0.001) (Table 4). On the same line, the rate of polyps was significantly higher at the age of 40–49 years than 18–39 years (217 (16.3%) and 65 (8.1%), respectively, *p* < 0.001) in which most of the cases detected were of single polyps in all groups. Similar to the first cohort, no remarkable differences were found in CRC prevalence among all age groups, *p* = 0.5 (Table 4).

#### Colonoscopy Findings in Males vs. Females

In total, 6582 females and 3542 males of all ages underwent colonoscopy with the indication of constipation alone. Normal colonoscopies were reported in 3613 (52.7%) women compared to 1455 (41%) males, *p* < 0.001 (Table S2). Higher rates of diverticulosis were found in males of all ages than in females of all ages (588(16.6%) vs. 784 (11.4%), *p* < 0.001) (Table S2), but no significant difference was found in patients under 40 years of age *p* = 0.15 (Table 5). Higher rates of polyps were found in males of all ages than females of all ages (1255 (35.4%) vs. 1676 (24.5%), *p* < 0.001, respectively) (Table S1). No significant differences were found between males and females under 40 years, *p* = 0.34 (Table 5). CRC was not found to be significantly different between males and females of all ages (14 (0.4%) and 19 (0.3%) patients respectively, *p* = 0.3) (Table S2). CRC was rarely found in females and males under 40 years (0.1% vs. 0, *p* = 0.5) (Table 5).

## 4. Discussion

Constipation is the most common gastrointestinal dysfunction, being more common in females and increasing with age [1,2,3]. In young females, functional constipation is more prevalent [14,15,16,17], while in older, secondary causes such as systemic disease, chronic medications, and structural abnormalities or obstructive findings are more common [6,8]. Despite this knowledge, many young women who have no alarm signs are referred to colonoscopies by gastroenterologists and general physicians as part of the diagnostic workup for gastrointestinal functional disorder. This large retrospective cohort included 11,910 colonoscopies performed in adults for an indication of constipation among other symptoms, and 10,394 colonoscopies that were performed for an indication of constipation alone showed higher rates of constipation in females than males within the same age groups (1:2 ratio). These findings align with results from other global studies that have investigated the epidemiological and gender differences in constipation prevalence. We found that constipation increased with age, as well as the number of colonoscopies increased steadily with age for this indication. The major findings of our study highlight the low diagnostic utility of performing colonoscopy among young women compared to older women, regardless of whether constipation was the sole indication or accompanied by other indications. We found that 75% of the colonoscopies of females under 40 years were normal in both cohorts, while significantly lower rates of normal exams were reported in other age groups. This finding comes in line with the recommendations of the ASGE and Seoul consensus, which argue against unwarranted routine colonoscopy with constipation as a sole indication due to similar outcomes as the asymptomatic population [19,20]. Importantly, the presence of additional symptoms such as abdominal pain or rectal bleeding in young women did not significantly alter the diagnostic outcomes in our cohort. Several studies revealed colonic pathological findings in 5–15% of patients with constipation including conditions like IBD, polyps, and diverticulosis but with few significant findings such as CRC. Popovic et al. and Ratnasingham et al. [21,22] reviewed the colonoscopy findings and failure rate in patients with constipation only and demonstrated the low yield of colonoscopy for this indication, regardless of age. They also highlighted a higher failure rate due to technical difficulties, poor preparation, and patient discomfort [21,22]. Our study reveals that the prevalence of conditions such as IBD, diverticulosis, and colorectal cancer (CRC) is relatively low among younger females presenting with constipation. While some studies suggest an association between constipation and colorectal pathologies, including CRC, our findings challenge the idea that constipation alone should warrant routine colonoscopy in young women. This is particularly true considering the low incidence of significant pathologies such as CRC in this cohort, even with accompanying symptoms. Our study showed results consistent with the findings of Abu Baker et al. [23] in which IBD diagnosis was found to be higher in constipated patients, particularly in subjects under 50 years (1.7% vs. 0.1%; *p* < 0.001). Our findings were also in concordance with James et al. who found that in 125 patients with UC (51% females), 58 (46%) fulfilled the definition of proximal constipation. The main symptoms were reduced stool frequency (69%), hard stools (43%), straining (24%), and sensation of incomplete emptying (14%). Proximal constipation was associated with female gender (OR 3.45 [1.45–8.24]), left-sided (OR 2.84 [1.14–7.11]), and concurrently active disease (OR 5.56 [1.96–16.67]) but not age, disease duration, or therapy [24]. In our cohort, IBD was the most significant finding in **Cohort 1** particularly in patients of young age, under 40 years old. The UC diagnosis was found to be more common than CD in all age groups. It is worth noting that the additional indications for colonoscopies in this cohort were abdominal pain (10%) and rectal bleeding (7.7%). The prevalence of IBD seems to be lower in young females in **Cohort 2**, who were referred for colonoscopy with the indication of constipation alone (<1% had IBD). Even though negligible, it was still significantly higher compared to older ages (over 50 years). The frequency rate of IBD was not significantly different between males and females in both cohorts of all ages. Regarding other relevant findings, in both cohorts, diverticulosis and polyp prevalence was dramatically lower in young females (18–39 years) compared to patients of older age, as well as compared to males across all age groups. Contrary to the common belief that diverticulosis is associated with constipation, Peery et al. showed more frequent bowel movements in individuals with diverticulosis, with a stronger association in participants with >10 colonic diverticula. In their study, there was no significant association between diverticulosis and altered bowel consistency [25]. Jarbink et al. showed that among 742 participants (54.6% women), 130 (17.5%) had diverticulosis; all participants with diverticulosis had sigmoid involvement, and they were more likely to report loose stools, urgency, passing mucus, and a high stool frequency [26]. In our study, we found a higher frequency rate of diverticular disease in older women with constipation. We have no data concerning the burden and the location of the diverticulosis in our cohort. In relation to constipation represented by polyps, in the study of Mohammed et al., 52 patients with colonoscopies out of 400 had polyps, 64% of them presented with rectal bleeding, and 42% presented with constipation. In addition, 30% of these patients had sigmoid polyps, where were of small size. There was no reference to age and gender [27]. In the study of Saad et al., out of 1852 patients over 65 years of age, 503 patients had polyps, 253 (50.3%) of whom were males. Twenty one percent of the clinical presentations of patients requiring colonoscopy was constipation. In addition, 61.8% were non-adenomatous polyps, 303 (60.2%) were minute, less than or equal to 5 mm, 138 (27.4%) were 6–9 mm, 45 (8.9%) were 10–20 mm, and 17 (3.4%) were more than 20 mm. No correlation was found between the clinical presentation and the polyp features [28]. In our study, colonic polyps were found in no more than 8% of young women under 40 years, and 70% of them were single polyps. We found very few cases of CRC in both cohorts in general and in the young group in particular, which underscores the finding that there should not be significant concern about missing significant life-threatening findings in this context. CRC is the most substantial outcome we looked for. The study of Abu Freha et al. on the same ethnic population showed in increase in early onset CRC in young patients under the age of 50 in the last decade (up to 9.8%), mainly among females [29]. Interestingly, in the current cohort, no significant differences were observed between all subgroups of age and gender presented with constipation. CRC rates were low among patients of all ages, in both males and females. One (0.1%) young female under 40 years of age was diagnosed with CRC, while 0.2–0.6% of those of older ages (greater than 50 years) were diagnosed with CRC in the two cohorts. This finding was remarkable and contrary to the expectations according to universal reports in which the CRC rate is significantly higher in older age, especially more in males than females presenting with constipation [30,31,32,33,34,35,36,37]. Our interpretation of this finding is that CRC cases diagnosed in our cohort did not have obstructive features, so constipation was not a hallmark even with associated signs and symptoms like rectal bleeding, abdominal pain, anemia, and weight loss. This further underscores the notion that CRC is not typically a concern in the diagnostic workup of constipation, particularly in younger women without alarming signs. The causal relationship between constipation and the risk of CRC was inconclusive and even contradictory in populations of different ethnicities. In the studies that found a positive relation, the prevalence of CRC was higher among females with constipation [38,39,40,41,42,43]. The strengths of this study lie in the nation-based design with a large cohort focusing on a population of young women who are largely investigated for one of the most common gastrointestinal disorders and are rarely addressed in literature, which enhances the generalizability and reliability of the observed abnormalities. However, our limitations include the retrospective nature of this analysis, the relatively small number of females under 40 years of age, and lack of data regarding polyps, such as size, localization, and histopathology.

## 5. Conclusions

The clinical utility of colonoscopies in the investigation of constipation in young females is not of a considerable diagnostic value. Clinical understanding should guide each case manager by using the correct beneficial diagnostic tools avoiding unnecessary invasive procedures.

## Figures and Tables

**Table 1:** Characteristics of Cohort 1.

7872 Females	Age Group 18–39 y *n* = 1017	Age Group 40–49 y *n* = 1535	Age Group 50–59 y *n* = 1972	Age 60 and Older *n* = 3348	
Age	29.79 ± 6.4	45.75 ± 2.9	54.73 ± 2.9	70.28 ± 15	
Cecal intubation	971 (95.5%)	1461 (95.2%)	1867 (94.7%)	3120 (93.2%)	0.005
**Chronic comorbidities**					
Diabetes mellitus	8 (0.8%)	41 (2.7%)	85 (4.3%)	369 (11%)	<0.001
Obesity	7 (0.7%)	14 (0.9%)	13 (0.7%)	23 (0.7%)	0.813
CIHD	1 (0.1%)	1 (0.1%)	3 (0.2%)	26 (0.8%)	<0.001
HTN	9 (0.9%)	69 (4.5%)	231 (11.7%)	981 (29.3%)	<0.001
Dyslipidemia	1 (0.1%)	16 (1%)	89 (4.5%)	272 (8.1%)	<0.001
Chronic renal failure	0	0	1 (0.1%)	7 (0.2%)	0.075
COPD	1 (0.1%)	3 (0.2%)	5 (0.3%)	15 (0.4%)	0.216
Asthma	24 (2.4%)	49 (3.2%)	58 (2.9%)	68 (2%)	0.055
NAFLD	1 (0.1%)	5 (0.3%)	6 (0.3%)	7 (0.2%)	0.618
**Additional indications**					
Abdominal pain	103 (10.1%)	70 (4.6%)	88 (4.5%)	125 (3.7%)	<0.001
FOBT	0	0	8 (0.4%)	17 (0.5%)	0.006
Rectal bleeding	78 (7.7%)	58 (3.8%)	48 (2.4%)	78 (2.3%)	<0.001
FH CRC	11 (1.1%)	28 (1.8%)	30 (1.5%)	26 (0.8%)	0.008
FH of polyps	1 (0.1%)	2 (0.1%)	1 (0.1%)	2 (0.2%)	0.815
Anemia	17 (1.7%)	35 (2.3%)	24 (1.2%)	49 (1.5%)	0.078
Screening	0	8 (0.5%)	28 (1.4%)	22 (0.7%)	<0.001
Weight loss	9 (0.9%)	6 (0.4%)	10 (0.5%)	24 (0.7%)	0.338

CIHD = Chronic Ischemic Heart Disease, HTN = Hypertension, COPD = Chronic Obstructive, Pulmonary Disease, NAFLD = Non-Alcoholic Fatty Liver Disease, FOBT = Fecal Occult Blood Test, FH = Family History, CRC = Colorectal Cancer.

**Table 2:** Colonoscopy Findings in All Age Groups—Cohort 1.

7872 Females	Age Group 18–39 y (1) *n* = 1017	Age Group 40–49 y (2) *n* = 1535	Age Group 50–59 y (3) *n* = 1972	Age 60 and Older (4) *n* = 3348	All	Group 1 vs. 2	Group 1 vs. 3	Group 1 vs. 4	Group 1 vs. 1 + 2 + 3
**Findings in colonoscopy**									
UC	12 (1.2%)	6 (0.4%)	2 (0.1%)	4 (0.1%)	<0.001	0.020	<0.001	0.004	<0.001
CD	7 (0.7%)	3 (0.2%)	0	0	<0.001	0.051	<0.001	0.072	<0.001
Angiodysplasia	0	0	2 (0.1%)	3 (0.1%)	0.489	N/A	0.310	0.203	0.389
Diverticulosis	5 (0.5%)	47 (3.1%)	159 (8.1%)	704 (21%)	<0.001	<0.001	<0.001	<0.001	<0.001
* Polyp of colon	75 (7.4%)	247 (16.1%)	442 (22.4%)	1125 (33.6%)	<0.001	<0.001	<0.001	<0.001	<0.001
CRC	1 (0.1%)	5 (0.3%)	5 (0.3%)	19 (0.6%)	0.103	0.246	0.369	0.432	0.117
Normal colonoscopy	761 (74.8%)	997 (65%)	1102 (55.9%)	1296 (38.75)	<0.001	<0.001	<0.001	<0.001	<0.001

UC = Ulcerative Colitis, CD = Crohn’s Disease, CRC = Colorectal Cancer. * One polyp (61.6%), two polyps (23.1%), and three or more polyps (15.3%) in all ages.

**Table 3:** Colonoscopy Findings in Young Males vs. Young Females—Cohort 1.

Females vs. Males Age 18–39	Females *n* = 1017	Males *n* = 476	*p*-Value
UC	12 (1.2%)	7 (1.5%)	0.641
CD	7 (0.7%)	2 (0.4%)	0.533
Angiodysplasia	0	1 (0.2%)	0.144
Diverticulosis	5 (0.5%)	7 (1.5%)	0.048
Polyp of colon	75 (7.4%)	54 (11.3%)	0.011
CRC	1 (0.1%)	0	0.494
Normal colonoscopy	761 (74.8%)	338 (71%)	0.119

UC = Ulcerative Colitis, CD = Crohn’s Disease, CRC = Colorectal Cancer.

**Table 4:** Colonoscopy Findings in All Age Groups—Cohort 2.

6852 Females	Age Group 18–39y (1) *n* = 807	Age Group 40–49 y (2) *n* = 1328	Age Group 50–59 y (3) *n* = 1739	Age 60 and Older (4) *n* = 2978	All	Group 1 vs. 2	Group 1 vs. 3	Group 1 vs. 4	Group 1 vs. 1 + 2 + 3
**Findings in colonoscopy**									
UC	6 (0.7%)	4 (0.3%)	2 (0.1%)	4 (0.1%)	0.008	0.147	0.008	0.003	0.001
CD	2 (0.2%)	3 (0.2%)	0	0	0.011	0.919	0.038	0.007	0.050
Angiodysplasia	0	0	2 (0.1%)	3 (0.1%)	0.513	0	0.335	0.367	0.414
Diverticulosis	3 (0.4%)	35 (2.6%)	132 (7.6%)	614 (20.6%)	<0.001	<0.001	<0.001	<0.001	<0.001
* Polyp of colon	65 (8.1%)	217 (16.3%)	396 (22.8%)	998 (33.5%)	<0.001	<0.001	<0.001	<0.001	<0.001
CRC	1 (0.1%)	4 (0.3%)	3 (0.2%)	11 (0.4%)	0.509	0.411	0.773	0.271	0.378
Normal colonoscopy	608 (75.3%)	863 (65%)	972 (55.9%)	1170 (39.3%)	<0.001	<0.001	<0.001	<0.001	<0.001

UC = Ulcerative Colitis, CD = Crohn’s Disease, CRC = Colorectal Cancer. * One polyp (61.5%), two polyps (22.6%), and three or more polyps (16.9%) in all age groups.

**Table 5:** Colonoscopy Findings in Young Males vs. Young Females—Cohort 2.

Females vs. Males Age 18–39	Females *n* = 807	Males *n* = 381	*p*-Value
UC	6 (0.7%)	5 (1.3%)	0.339
CD	2 (0.2%)	2 (0.5%)	0.442
Angiodysplasia	0	1 (0.3%)	0.145
Diverticulosis	3 (0.3%)	4 (0.4%)	0.154
Polyp of colon	65 (8.1%)	37 (9.7%)	0.341
CRC	1 (0.1%)	0	0.491
Normal colonoscopy	608 (75.3%)	275 (72.2%)	0.244

UC = Ulcerative Colitis, CD = Crohn’s Disease, CRC = Colorectal Cancer.

## Data Availability

The original contributions presented in this study are included in the article/Supplementary Materials. Further inquiries can be directed to the corresponding author.

## Supplementary Materials

The following supporting information can be downloaded at: https://www.mdpi.com/article/10.3390/diagnostics15101209/s1, Table S1: Colonoscopy Findings in Males vs. Females of All Ages-Cohort 1; Table S2: Colonoscopy Findings in Males vs. Females of All Ages-Cohort 2.

## Abbreviations

The following abbreviations are used in this manuscript:

IBD	Inflammatory bowel disease
UC	Ulcerative colitis
CD	Crohn’s disease
CRC	Colorectal cancer
IBS	Irritable bowel syndrome
FOBT	Fecal occult blood test
