# Supplementary material for: Clinical Yield of Colonoscopy in Evaluation of Young Women with Constipation: An Age- and Gender-Based Analysis
Source: Diagnostics (Basel). 2025 May 11;15(10):1209. doi: 10.3390/diagnostics15101209 (PMC12110052; doi:10.3390/diagnostics15101209)
Supplement: Supplementary file 1 [file diagnostics-15-01209-s001.zip › diagnostics-3590495-supplementary.pdf]

Supplementary Material

Table S1: Colonoscopy Findings in Males vs. Females of All Ages- Cohort 1

**Females vs males All age groups**	**Females n=7872**	**Males n=4038**	**p-value**
UC	24 (0.3%)	12 (0.3%)	0.942
CD	10 (0.1%)	4 (0.1%)	0.673
Angiodysplasia	5 (0.1%)	4 (0.1%)	0.504
Diverticulosis	915 (11.6%)	656 (16.2%)	<0.001
Polyp of colon	1889 (24%)	1417 (35.1%)	<0.001
CRC	30 (0.4%)	18 (0.4%)	0.598
Normal colonoscopy	4156 (52.8%)	1687 (41.8%)	<0.001

UC= Ulcerative Colitis, CD =Crohn's Disease, CRC= Colorectal Cancer

Table S2: Colonoscopy Findings in Males vs. Females of All Ages- Cohort 2

**Females vs males All age groups**	**Females n=6852**	**Males n=3542**	**p-value**
UC	16 (0.2%)	8 (0.2%)	0.939
CD	5 (0.1%)	3 (0.1%)	0.838
Angiodysplasia	5 (0.1%)	1 (0.1%)	0.759
Diverticulosis	784 (11.4%)	588 (16.6%)	<0.001
Polyp of colon	1676 (24.5%)	1255 (35.4%)	<0.001
CRC	19 (0.3%)	14 (0.4%)	0.311
Normal colonoscopy	3613 (52.7%)	1455 (41.1%)	<0.001

UC= Ulcerative Colitis, CD =Crohn's Disease, CRC= Colorectal Cancer
